# Toothbrush-Dentifrice Abrasion of Dental Sealants: An *In Vitro* Study

**DOI:** 10.1055/s-0041-1735798

**Published:** 2021-12-02

**Authors:** Angkhana Sangpanya, Pornpoj Fuangtharnthip, Vanida Nimmanon, Praewpat Pachimsawat

**Affiliations:** 1 Department of Periodontology, Faculty of Dentistry, Khon Kaen University, Khon Kaen, Thailand; 2 Department of Advanced General Dentistry, Faculty of Dentistry, Mahidol University, Bangkok, Thailand

**Keywords:** abrasion, dental sealants, dentifrice, toothbrushing

## Abstract

**Objective** This study sought to investigate the toothbrush-dentifrice abrasion of dental sealants.

**Materials and Methods** Weight loss (∆W) and depth loss (∆D) were used as abrasion indicators. Sealant samples from nine products were soaked in dentifrice slurry and abraded by using a toothbrushing machine with a brushing force of 300 g. The mean percentages of ∆W and mean values of ∆D after 24,000 and 48,000 strokes of brushing were compared by using paired *t* -test. A comparison of these mean values among sealant products was performed by using one-way ANOVA and multiple comparison analysis (Scheffe's test).

**Results** Abrasive wear was observed in all sealants. Teethmate F-1 (Kuraray Noritake, Tokyo, Japan)—a fluoride-releasing unfilled sealant—exhibited the maximum abrasive wear, with ∆W and ∆D values of 1.14% ± 0.37% and 12.84 ± 4.28 µm, respectively. Delton (Dentsply Sirona, Charlotte, North Carolina, United States), a light-cured unfilled sealant, showed the minimum abrasive wear, with ∆W and ∆D values of 0.41% ± 0.09% and 2.93 ± 1.23 µm, respectively. No statistical differences were observed among unfilled sealants except when compared with Teethmate F-1. Similarly, no differences were observed when comparing among filled sealants and flowable composite.

**Conclusion** Abrasive wear occurred in all sealants after brushing with dentifrice. Almost all unfilled sealants showed less wear compared with both filled sealants and flowable composite. However, the low abrasive values of all sealants after brushing with dentifrice implied that there is no clinical significance to this finding.

## Introduction

Dental sealant has been accepted as a material in preventing tooth decay. 1 The effective area of prevention is at the pit and fissure, where toothbrush bristles cannot reach and clean thoroughly. 2 The most popular sealants are resin based that consist of four basic components: resin, filler, activator, and initiator. 3 Resins, the fundamental substance of sealants, are mostly aromatic dimethacrylates such as bisphenol A-glycidyl methacrylate (Bis-GMA) and aliphatic dimethacrylates such as urethane dimethacrylate (UDMA) and triethylene glycol dimethacrylate (TEGDMA). 4 Fillers such as quartz, silica, and glass particles are added to the resin base to increase the strength and wear resistance of the sealant. 5 An activator provides energy to activate an initiator to generate free radicals and initiate polymerization. There are two types of polymerization: autopolymerization or chemically-activated (self-cure) and photopolymerization (light-cure). 6 For autopolymerization, benzoyl peroxide and organic amine are used as initiator and activator, respectively. 7 While in photopolymerization, organic amine and ∝-diketones such as camphorquinone are used as initiator and visible light of 430 to 490 µm is used as an activator. 4

Resin sealants may be classified into four types depending on the amount of filler and the presence or absence of a fluoride-releasing property. 3 8 Unfilled sealants or conventional sealants range from having no filler to less than 30% by weight of filler, while the filler contents of filled sealants range from 30 to 50% by weight. 4 9 Flowable composites are a modification of composite resins suggested to be used as a sealant. 10 This material is created by reducing the amount of filler and increasing the resin content to make the material less viscous and improve its penetration capacity. 11 Flowable composites contain 50 to 70% filler by weight. 12 The last type of resin sealant is fluoride-releasing sealant, which comes in both unfilled and filled varieties. 13 Fluoride is either added to unpolymerized resin or chemically bound to resin and the sealant is expected to release fluoride after the materials set to improve caries prevention. 14

Dental sealant longevity depends upon the amount of overlying sealant retained in pits and fissures. 15 Sealant is retained on primary and permanent molars at a retention rate of approximately 70% at 3 years after placement. 16 The loss of sealant appears to result from two aspects: inadequate retention and wear. 17 Abrasive wear is an important factor that occurs in the mouth from chewing food and brushing the teeth. 18 The severity of toothbrushing abrasion depends upon the shape and particle size of abrasive agents in the toothpaste and the stiffness of the toothbrush bristles. 19 20

Various parameters such as the measurement of weight loss and vertical loss or depth loss in dental materials in relation to toothbrush-dentifrice abrasion have been assessed. 19 21 Some studies suggested most abrasion in terms of volume loss, depth loss, and area loss of sealants occurred in the first 6 months, depending on the physical attributes of the teeth. 22 23 Another 2-year clinical abrasion study reported no statistical difference existed between filled and unfilled sealants. 24 In contrast, an in vitro abrasion study conducted by the application of abrasive paper showed that unfilled sealants were abraded at twice the rate of the filled sealants, but the abrasion rate was reduced to one-half or one-third when more than 50% by weight of fillers was added. 25 Moreover, the greater the amount of bonding agent added to dilute the composite resin, the higher is the abrasion rate. This indicated broadly that the abrasive resistance increases when more inorganic filler is added. 26 The effects of abrasive wear in dental sealants include a decrease in their quantity, the onset of marginal breakdown, and the loss of sealant, all of which reduce sealant longevity. 25 Variations in polymerization characteristics, color, and price are obvious details required by clinicians to be able to select the appropriate sealant. In fact, the physical properties and clinical performance of sealant should be offered together when selecting the suitable sealant to use. 5 The present study aimed to investigate toothbrush-dentifrice abrasion of various types of sealants in an effort to provide useful basic information to assist clinicians with sealant selection.

## Materials and Methods

### Sealants Preparation

Nine different dental sealants were examined ( Table 1 ). These sealants contain different amounts of fillers and polymerize either by self- or light-curing. The filler levels included were less than 30% in the unfilled sealant (U), 30 to 50% in the filled sealant (F), and more than 50% in the flowable composite (FC). Among these, TF, HF, and UP were also fluoride-releasing sealants. For each sealant, two studies of sealant abrasion were conducted to collect measurements of weight loss and depth loss. About 36 pieces of sealant were prepared, resulting in a total of 324 samples. The samples were prepared on a split Teflon mold (3 mm thick × 6 mm inside diameter) with a Dental Mylar strip (Mytrip; Dentamerica, Industry, California, United States) and a glass plate. They were fixed with a compressive instrument. Samples were cured by using a light-curing unit (XL 3000; 3M ESPE, St Paul, Minnesota, United States), except for CS and DS, which were self-cured. Sealant samples were removed from the mold and all excess margins were trimmed using a number 11 surgical blade without touching the above and beneath surfaces. For the depth loss study, two marks were made with a permanent marker on the sides of the samples, opposite each other. These marks were used as reference lines to locate the exact positions in the sample holders. All prepared samples were stored in an incubator (Memmert, Büchenbach, Germany) and were soaked in distilled water at 37°C for 15 days.

**Table 1:** Sealant products examined

Sealant product: polymerization	Product abbreviation	Monomer	Filler (%)	Manufacturer
Concise: LC	CL	Bisphenol A diglycidylether dimethacrylate TEGDMA	Silica (5–10); U	3M ESPE, St Paul, MN, USA
Concise: SC	CS	Bisphenol A diglycidylether dimethacrylate TEGDMA	Silica (5–10); U	3M ESPE, St Paul, MN, USA
Delton: LC	DL	Aromatic and aliphatic dimethacrylate	Silica (5.4); U	Dentsply Sirona, Charlotte, NC, USA
Delton: SC	DS	Aromatic and aliphatic dimethacrylate	None (0); U	Dentsply Sirona, Charlotte, NC, USA
Dentguard: LC	DG	Bis-GMA TEGDMA UDMA	Silica (4–5); U	National Metal and Materials Technology Center (MTEC), Thailand
Teethmate F-1: LC	TF	TEGDMA Hydrophobic dimethacrylate 2-Hydroxyethyl methacrylate 10-Methacryloyloxydecyl dihydrogen phosphate Methacryloyl fluoride-methyl methacrylate copolymer	None (0); U	Kuraray Noritake, Tokyo, Japan
Helioseal F: LC	HF	Bis-GMA TEGDMA UDMA	Fluorosilicate glass Silica dioxide (40.5); F	Ivoclar Vivadent AG, Liechtenstein
Sealite: LC	SL	Bis-GMA TEGDMA	Ground glass (40); F	KERR Manufacturing Company, Orange, CA, USA
Ultraseal XT plus: LC	UP	Bis-GMA TEGDMA Diurethane dimethacrylate	Glass ionomer glass (60); FC	Ultradent Products Inc., South Jordan, UT, USA

Abbreviations: Bis-GMA, bisphenol A-glycidyl methacrylate; F, filled sealant; FC, flowable composite; LC, light-cured polymerization; SC, self-cured polymerization; TEGDMA, triethylene glycol dimethacrylate; U, unfilled sealant; UDMA, urethane dimethacrylate.

### Abrasive Procedure by Toothbrushing Machine

The toothbrushing machine was set at 300 strokes/minute. All samples were brushed for 48,000 strokes and one stroke of brushing equaled being brushed one time (forward or backward). A new toothbrush head (soft bristles; AIM, Nonthaburi, Thailand), and the dentifrice slurry were changed every 12,000 brushing strokes. The load was 300 g and the dentifrice slurry was a mixture of artificial saliva, distilled water, and toothpaste (Colgate Great Regular Flavor; Colgate-Palmolive Ltd., Bangkok, Thailand) at a ratio of 1:1:2 (50 mL:50 mL:100 g). The abrasive in this toothpaste was dicalcium phosphate dihydrate, and the other ingredients were sodium monofluorophosphate, sodium lauryl sulfate, water, glycerin, carboxymethylcellulose sodium, sodium pyrophosphate, and sodium saccharin. 27

**Fig. 1:**
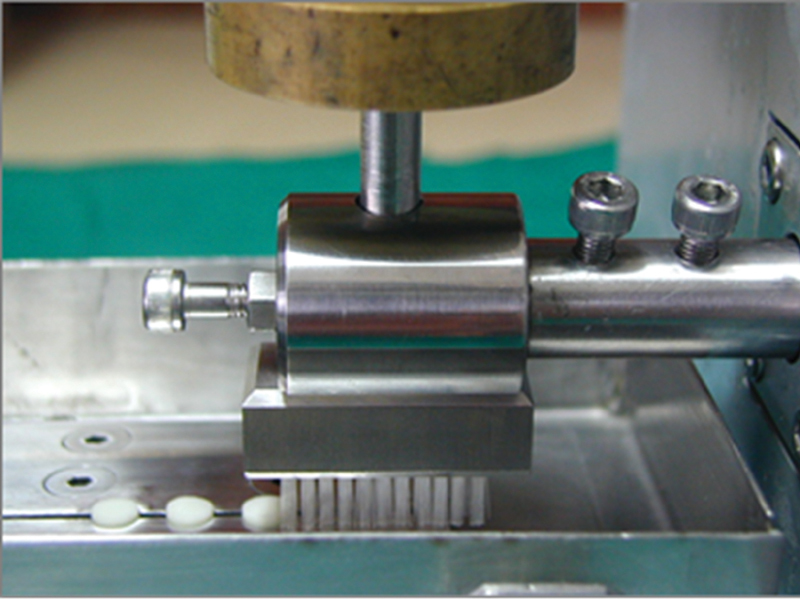
The toothbrushing machine and the sealants that were installed.

Three different sealant samples were randomly chosen and placed on the removable stage of the toothbrushing machine ( Fig. 1 ). These samples were fastened, and their surfaces were arranged to equally emerge by 1 mm from the horizontal plane of the stage. For the depth loss study, on the upper surface of each sample at both sides, reference lines were marked at 1.25-mm off the margin using a number 11 surgical blade. These reference lines were then masked with polyvinyl chloride tape (50 μm thick, TIP TAPE Supbumphenboon Ltd., Thailand), and the sample surface revealed a 1.5-mm wide track parallel to the brushing direction. These masking areas were unabraded and used as baselines to compare with the abrasive area.

Before measurement, the pieces of polyvinyl chloride tape were removed (depth loss study). The samples were then rinsed under running tap water for approximately 30 seconds and shaken by the ultrasonic cleaner (Vibraclean 300 ultrasonic cleaner, MDT Harvey, United States) for 5 minutes. Thereafter, the samples were blotted dry by using a paper towel and left at room temperature for 30 minutes.

### Weight Loss Measurement

The percentage of weight loss was calculated by comparing the weight of the sample before and after brushing for 24,000 strokes (∆W _24,000_ ) and after brushing 48,000 strokes (∆W _48,000_ ). For each measurement, one sample was weighed three times 20 seconds apart by using an analytical balance (Precisa Model 262 SMA-FR; Precisa Instruments AG, Zurich, Switzerland). The mean weight was determined for each sealant.

### Depth Loss Measurement

The removable stage holding samples were placed on the adjustable table of the surface roughness tester (Taylor Hobson Form Talysurf series 2, Rank P.T.O., Cornor's Company, England). Acrylic jigs were used to place the stage in the same position for each measurement. The surface profile of each sample was performed by using a tracing stylus measuring 2 μm in diameter. Tracing areas (lines) were performed at three positions perpendicular to the brushing direction and reference line. Each tracing line was 1.5 mm apart and 4 mm in distance and had to pass both reference lines. The tracing speed was 1 mm/second.

**Fig. 2:**
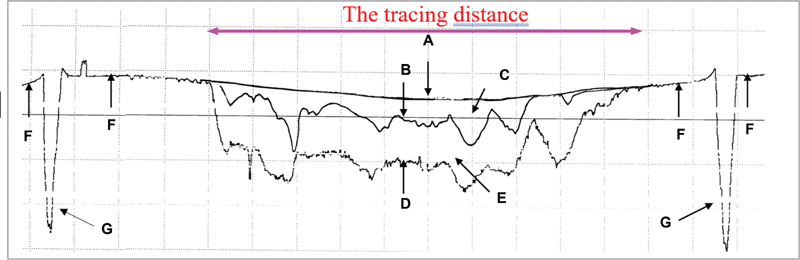
The surface profile in each position, with the “after” photograph superimposed onto the original one. ( **A** ) surface profile before brushing, ( **B** ) surface profile after brushing for 24,000 strokes, ( **C** ) area of wear after brushing for 24,000 strokes, ( **D** ) surface profile after brushing for 48,000 strokes, ( **E** ) area of wear after brushing between 24,000 and 48,000 strokes, (C) + (E) area of wear after brushing for 48,000 strokes, ( **F** ) line in no-brushing areas, ( **G** ) reference lines.

The printout of the surface profiles in each position was used for comparison with the original surface profile measured before the abrasive procedure by superimposing the latter onto the original. The area between these two profiles was calculated by using the Image Pro Plus version 3.01.00 software program (Media Cybernetics, Rockville, Maryland, United States), and the depth loss of each position was calculated (depth loss area divided by the tracing distance). The average depth loss in three tracing positions was used as the mean depth loss in each measurement. Specifically, ∆D _24,000_ was depth loss after brushing 24,000 strokes or area (C)/the tracing distance in Fig. 2 , ∆D _24,000–48,000_ was depth loss between brushing 24,000 and 48,000 strokes or area (E)/the tracing distance in Fig. 2 , and ∆D _48,000_ was depth loss after brushing 48,000 strokes or area (C + E)/the tracing distance in Fig. 2 .

### Statistical Analysis

All data were analyzed for normal distribution by using the Kormogorov–Smirnov test. When the data were not normally distributed, they were transformed to square roots (weight loss study) and based-e logarithm (depth loss study). Furthermore, Levene's test was used to analyze the homogeneity of variance. Paired *t* -test was used to compare the mean percentages of weight loss at ∆W _24,000_ versus ∆W _48,000_ and mean depth loss at ∆D _24,000_ versus ∆D _48,000_ of each sealant product. One-way ANOVA and multiple comparisons were used to compare ∆W and ∆D among sealants at each time interval. For multiple comparisons, the Scheffe's test was used. Statistical analyses were performed at *p* ≤0.01.

## Result

### Weight Loss

**Fig. 3:**
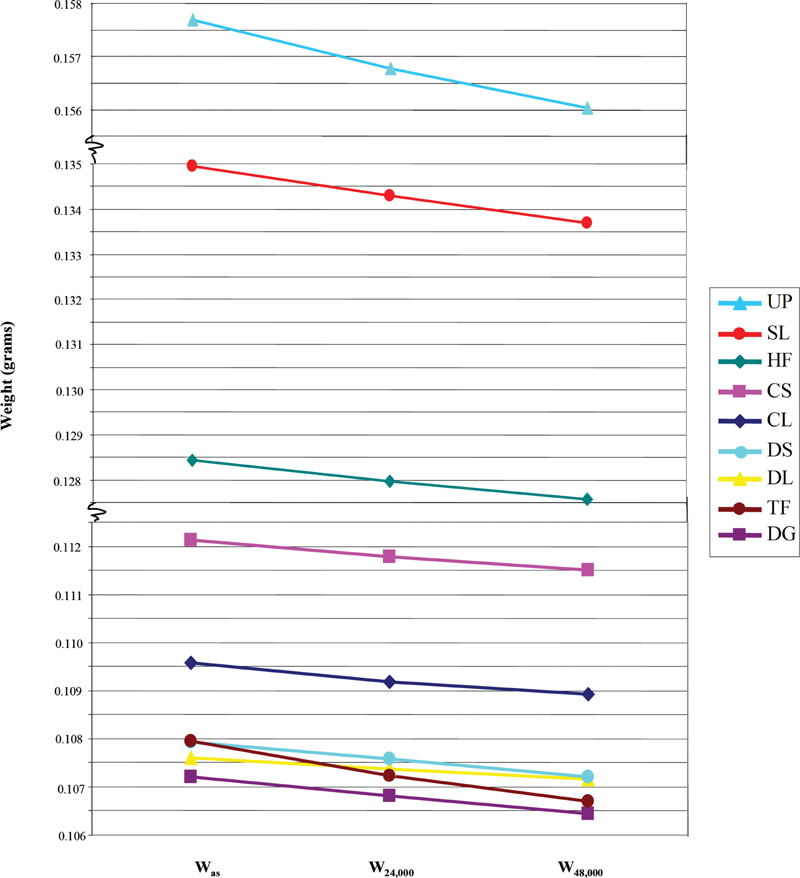
Mean weights of each sealant after soaking (W _as_ ), following brushing for 24,000 (W _24,000_ ) or 48,000 strokes (W _48,000_ ).

Weight loss after brushing was found to have occurred with all sealants, with the degree of loss corresponding to the number of times of brushing ( Fig. 3 ). TF had the maximum weight loss. As shown in Table 2 , the percentage of weight loss of TF at ∆W _24,000_ and ∆W _48,000_ were 0.66 and 1.14%, respectively. Second and third in line in terms of the most weight loss were UP and SL, with percentages of weight loss at ∆W _24,000_ and ∆W _48,000_ being 0.58% and 1.04% for UP and 0.49 and 0.93% for SL, respectively. Meanwhile, DL showed the minimum weight loss; its percentages of weight change at ∆W _24,000_ and ∆W _48,000_ were 0.23 and 0.41%, respectively, which were significantly different from TF and UP.

**Table 2:** Mean percentages of weight loss among nine sealants

Sealants	Mean percentages of weight loss					
	∆W _24,000_		∆W _24,000–48,000_		∆W _48,000_	
	% (SD)	Sig. Diff. a	% (SD)	Sig. Diff. a	% (SD)	Sig. Diff. a
CL	0.35 (0.41)		0.24 (0.08)	TF, SL, UP	0.59 (0.43)	TF, UP
CS	0.33 (0.10)		0.23 (0.10)	TF, SL, UP	0.55 (0.08)	TF, UP
DL	0.23 (0.08)	TF, UP	0.19 (0.05)	DS, DG, TF, SL, UP	0.41 (0.09)	TF, SL, UP
DS	0.33 (0.10)		0.35 (0.10)	DL	0.68 (0.12)	TF
DG	0.37 (0.14)		0.35 (0.07)	DL	0.72 (0.15)	
TF	0.66 (0.32)	DL	0.49 (0.10)	CL, CS, DL	1.14 (0.37)	CL, CS, DL, DS, HF
HF	0.38 (0.23)		0.31 (0.08)		0.69 (0.25)	TF
SL	0.49 (0.08)		0.45 (0.05)	CL, CS, DL	0.93 (0.09)	DL
UP	0.58 (0.14)	DL	0.46 (0.16)	CL, CS, DL	1.04 (0.26)	CL, CS, DL

Abbreviations: CL, Concise light-cured; CS, Concise self-cured; DG, Dentguard; DL, Delton light-cured; DS, Delton self-cured; HF, Helioseal F; SL, Sealite; TF, Teethmate F-1; UP, Ultraseal XT plus.

a *p* <0.01 compared with the one on the first column.

### Depth Loss

After brushing for 24,000 and 48,000 strokes, the depth loss was increased in all sealants in proportion to the increased number of strokes. Significant differences in depth loss were observed in all sealants when compared between brushing for 24,000 (∆D _24,000_ ) and 48,000 (∆D _48,000_ ) strokes ( *p* < 0.01). As shown in Table 3 , TF had the maximum value of depth loss (6.50 μm for ∆D _24,000_ and 12.84 μm for ∆D _48,000_ ), followed by UP (5.18 μm for ∆D _24,000_ and 10.02 μm for ∆D _48,000_ ). In contrast, DL had the minimum value of depth loss (1.72 μm for ∆D _24,000_ and 2.93 μm for ∆D _48,000_ ), which was significantly different from that of TF.

**Table 3:** Depth loss among nine sealants

Sealants	Depth loss (µm)					
	∆D _24,000_		∆D _24,000–48,000_		∆D _48,000_	
	Mean (SD)	Sig. Diff a	Mean (SD)	Sig. Diff a	Mean (SD)	Sig. Diff a
CL	2.35 (1.50)	TF	1.91 (1.45)		4.26 (2.52)	TF
CS	2.06 (1.12)	TF	2.51 (2.69)		4.56 (3.52)	TF
DL	1.72 (0.86)	TF, UP	1.21 (0.76)	TF	2.93 (1.23)	TF, UP
DS	4.33 (2.50)		4.18 (4.66)		8.51 (6.70)	
DG	2.41 (1.41)		2.73 (3.39)		5.14 (4.23)	
TF	6.50 (2.71)	CL, CS, DL	6.34 (3.14)	DL	12.84(4.28)	CL, CS, DL
HF	4.36 (2.42)		3.48 (2.24)		7.84 (4.09)	
SL	3.65 (1.94)		3.55 (2.29)		7.19 (3.85)	
UP	5.18 (2.04)	DL	4.84 (2.04)		10.02 (2.87)	DL

Abbreviations: CL, Concise light-cured; CS, Concise self-cured; DG, Dentguard; DL, Delton light-cured; DS, Delton self-cured; HF, Helioseal F; SL, Sealite; TF, Teethmate F-1; UP, Ultraseal XT plus.

a *p* <0.01 compared with the one on the first column.

Generally, this study demonstrated that abrasive wear occurred in all sealants as measured by weight loss and depth loss. No statistical differences were observed among the unfilled sealants except as compared with TF. Also, no differences were observed when comparing the filled sealants and flowable composites. Almost all unfilled sealants showed less wear relative to both the filled sealants and the flowable composites.

## Discussion

Various means have been used to measure the wear of resin-based dental materials. 28 29 30 31 This present study chose weight loss and depth loss as parameters to verify the nature of toothbrush-dentifrice abrasive wear of nine dental sealants with differences in fillers, types of polymerization, and fluoride incorporation. A toothbrushing machine that simulated back and forth brushing strokes was applied at different brushing forces and frequencies, including the duration of brushing time. To resemble toothbrushing in the oral cavity, dentifrice and artificial saliva were included. Colgate (Great Regular Flavor), having low radioactive dentin abrasion (RDA = 40), was selected to be a dentifrice and mixed with artificial saliva and distilled water at the ratio of 2:1:1. This slurry mixture was similar to that used in the studies by Tanoue et al 21 and Kanter et al. 29 On the contrary, some studies have employed only dentifrice and distilled water at the ratio of 1:1 as soaking slurry. 12 32 The choice of the slurry mixture depends on the purpose of the study on which factor to investigate.

For the abrasive procedure, the toothbrushing machine was set at the brushing frequency of 300 strokes/minute to simulate brushing speed on the occlusal surface 30 and a brushing duration at 24,000 and 48,000 strokes to represent 2 and 4 years of brushing duration. 33 In addition, the 300-g weight applied on an experimental toothbrush represented the average force that a 30-year-old adult would normally produce when brushing. 34 The AIM toothbrush was chosen due to its flexible and round-ended bristles characteristics that enhance teeth cleanliness with less gingival abrasion. 35 However, the soft-bristle toothbrush can be more abrasive on acrylic material than hard-bristle toothbrushes in the presence of dentifrice. 20

After undergoing the abrasive procedure conducted by the toothbrushing machine, all sealants showed both weight loss and depth loss. These parameters demonstrated correlated results and similar directions in terms of abrasive wear. Concomitantly, material solubility and water sorption may occur as well. 36 The results revealed that TF, an unfilled sealant with a fluoride-releasing property, experienced the maximum weight loss, which corresponded to the maximum depth loss after brushing, in comparison with the other sealants. This indicated that TF had the highest abrasive wear in the present study. As the main matrix resin of TF was TEGDMA, such may have supported the material to become more soluble in water and show enhanced weight loss. 36 Kawai et al previously found that wear resistance increased with greater content of TEGDMA. 28 With respect to minimal wear, DL, another unfilled sealant, achieved all the minimum values for both weight loss and depth loss. Therefore, DL would be expected to show the least wear and greater abrasive resistance as compared with the other sealants.

With respect to the type of polymerization, light-cured sealants (CL and DL) showed no statistical significance in abrasion values in terms of both weight loss and depth loss compared with their conventional self-cured sealants (CS and DS). Regarding the degree of conversion, polymeric resin formed by a light-activated process (light-curing) does not differ in the degree of conversion relative to chemically activated (self-curing) resin if they contain the same monomer formulation as long as adequate light-curing is employed. Since wear resistance positively correlates with the degree of conversion, light-cured sealant and their self-cured counterparts showed no difference with respect to abrasive wear. 3

This study demonstrated that flowable composite and filled sealants exhibited higher abrasive wear in comparison with unfilled sealants. This is in agreement with the findings of Jensen et al 24 who studied sealant wear by tooth replica technique, and those of Roberts et al who used an in vitro two-body abrasion. 37 In contrast, other two-body abrasion studies performed using silicon carbide paper demonstrated that unfilled sealants had twice the abrasive wear as compared with filled sealants did and three to six times that as compared with composite resins. 38 On the other hand, previous studies have shown that filled and unfilled sealants exhibited similar retention rates. 39 40

When comparing the three fluoride-releasing sealants, TF showed the maximum abrasive wear, followed by UP and HF. In consideration of the way fluoride is incorporated into the sealant composition, two different methods were adopted. HF and UP involved soluble fluoride salts added to unpolymerized resins, whereas TF involved organic fluoride compounds chemically bound to resin. The former system of fluoride release occurred by water diffused in the hydrophobic matrix, which dissolved the hydrophilic fluoride ion and then diffused out of the sealant matrix into the surrounding environment. Notably, this fluoride-release technique might weaken the sealant's surface. 41 The latter system was an ion exchange system, where fluoride was released by exchanging with other ions. In this context, there should not be any significant decrease in the sealant's strength. However, even though the fluoride-incorporation method of TF was better, its abrasive resistance was worse than that of both HF and UP.

In the depth loss study, the maximum volume loss of sealant was 12.84 µm after brushing for 48,000 strokes, which corresponds to 4 years of brushing. 33 In a previous in vivo study, the mean value of the maximum depth loss among the sealants tested was 221.8 µm after 30 months. 22 This was due to the fact that, in the oral cavity, not only brushing, but also the type of food eaten and the pattern of chewing contributed to increased abrasion. The clinical application based on the current study was that abrasive wear from brushing had a lesser effect on sealant abrasion and should be considered as a minor factor when evaluating the physical properties or longevity of sealant. The limitations of this study were that only one brand of flowable composite was tested, and that only abrasive wear was examined. The erosive wear could also play a role as mentioned, and this should be investigated in the future. However, this point was also the strength of the current study, where we studied only the effect of abrasive wear from toothbrush-dentifrice without the interference of erosive wear in a real situation.

## Conclusion

Abrasive wear from toothbrushing occurred with all sealants and corresponded to brushing time intervals. Almost all unfilled sealants showed less wear as compared with filled sealants and flowable composites. However, the lower values of toothbrush-dentifrice abrasion among sealants implied minor influence on the clinical wear of sealants occurring in the oral cavity.
